# Crystal structure of 3-bromo-2-hy­droxy­benzo­nitrile

**DOI:** 10.1107/S2056989015011974

**Published:** 2015-06-27

**Authors:** Sean R. Dickinson, Peter Müller, Joseph M. Tanski

**Affiliations:** a Department of Chemistry, Vassar College, Poughkeepsie, NY 12604, USA; b X-Ray Diffraction Facility, MIT Department of Chemistry, 77 Massachusetts Avenue, Building 2, Room 325, Cambridge, MA, 02139-4307, USA

**Keywords:** crystal structure, disorder, hydrogen bonding, π-stacking

## Abstract

The crystal structure of the title compound, C_7_H_4_BrNO, has been determined, revealing a partial mol­ecular packing disorder such that a 180° rotation of the mol­ecule about the phenol C—O bond results in disorder of the bromine and nitrile groups. The disorder has been parameterized as a disorder of only the bromine and nitrile substituents on a unique phenol ring. An intra­molecular O—H⋯Br contact occurs. In the crystal, O—H⋯Br/O—H⋯N_nitrile_ hydrogen bonding is present between the disordered bromine and nitrile substituents and the phenol group, forming a spiral chain about a twofold screw axis extending parallel to the *b-*axis direction. Within this spiral chain, the mol­ecules also inter­act, forming offset face-to-face π-stacking inter­actions with plane-to-centroid distance of 3.487 (1) Å.

## Related literature   

For syntheses of the title compound, see: Anwar & Hansen (2008 ▸); Nakai *et al.* (2014 ▸); Whiting *et al.* (2015 ▸). For its use as a synthetic reagent, see: Li & Chua (2011 ▸); Mulzer & Coates (2011 ▸). For related crystal structures, see: Beswick *et al.* (1996 ▸); Oh & Tanski (2012 ▸). For information on π-stacking, see: Hunter & Sanders (1990 ▸); Lueckheide *et al.* (2013 ▸). For information on the refinement of disordered crystal structures, see: Müller (2009 ▸); Thorn *et al.* (2012 ▸).
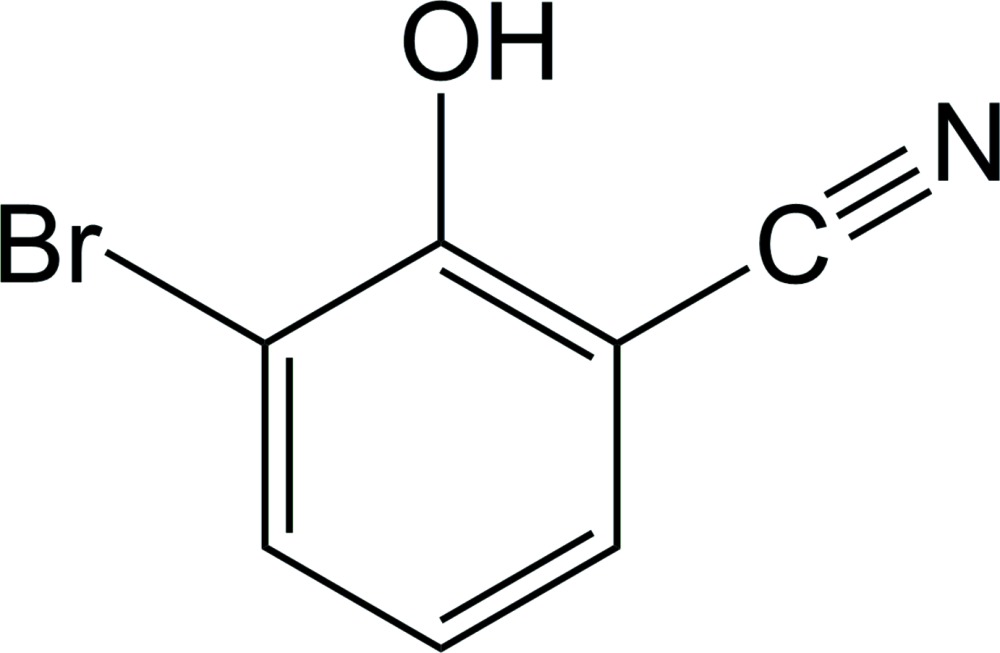


## Experimental   

### Crystal data  

C_7_H_4_BrNO
*M* *_r_* = 198.02
Monoclinic, 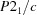

*a* = 13.0171 (7) Å
*b* = 3.8488 (2) Å
*c* = 13.5989 (7) Å
β = 96.062 (1)°
*V* = 677.50 (6) Å^3^
*Z* = 4
Mo *K*α radiation
μ = 5.98 mm^−1^
*T* = 125 K
0.22 × 0.10 × 0.04 mm

### Data collection  

Bruker APEXII CCD diffractometer
Absorption correction: multi-scan (*SADABS*; Bruker, 2013 ▸) *T*_min_ = 0.57, *T*_max_ = 0.80
9903 measured reflections
1977 independent reflections
1776 reflections with *I* > 2σ(*I*)
*R*_int_ = 0.025

### Refinement  

*R*[*F* ^2^ > 2σ(*F* ^2^)] = 0.020
*wR*(*F* ^2^) = 0.049
*S* = 1.08
1977 reflections
110 parameters
102 restraints
H atoms treated by a mixture of independent and constrained refinement
Δρ_max_ = 0.37 e Å^−3^
Δρ_min_ = −0.45 e Å^−3^

### 

Data collection: *APEX2* (Bruker, 2013 ▸); cell refinement: *SAINT* (Bruker, 2013 ▸); data reduction: *SAINT*; program(s) used to solve structure: *SHELXS97* (Sheldrick, 2008 ▸); program(s) used to refine structure: *SHELXL2014* (Sheldrick, 2015 ▸); molecular graphics: *SHELXTL2014*; software used to prepare material for publication: *SHELXTL2014*, *OLEX2* (Dolomanov *et al.*, 2009 ▸) and *Mercury* (Macrae *et al.*, 2008 ▸).

## Supplementary Material

Crystal structure: contains datablock(s) global, I. DOI: 10.1107/S2056989015011974/ld2134sup1.cif

Structure factors: contains datablock(s) I. DOI: 10.1107/S2056989015011974/ld2134Isup2.hkl

Supporting information file. DOI: 10.1107/S2056989015011974/ld2134Isup3.cml

**Fig. 1.**
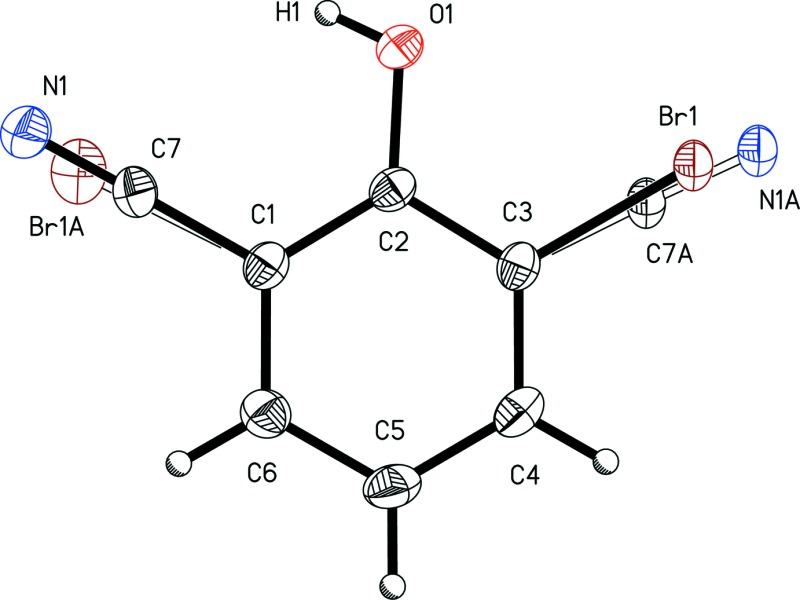
: A view of the title compound showing the disordered nitrile and bromine substituents, with displacement ellipsoids shown at the 50% probability level.

. DOI: 10.1107/S2056989015011974/ld2134fig1.tif
A view of the title compound showing the disordered nitrile and bromine substituents, with displacement ellipsoids shown at the 50% probability level.

**Fig. 2.**
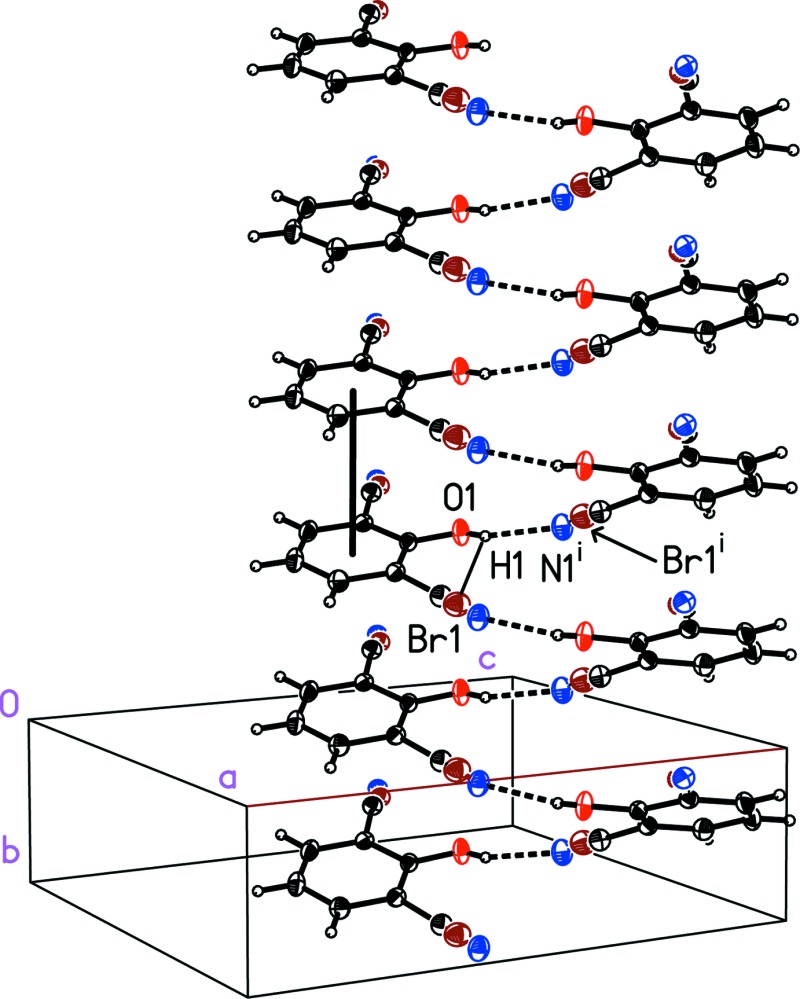
: A view of the intermolecular O—H···Br/O—H···Nnitrile hydrogen bonding interactions (dashed lines) forming a helical one-dimensional chain, with displacement ellipsoids shown at the 50% probability level. See Table 1 for symmetry code (i). A thin solid line indicates an intramolecular O—H···Br hydrogen bond, and a thick solid line indicates a π-stacking centroid-to-centroid interaction.

O H O H nitrile . DOI: 10.1107/S2056989015011974/ld2134fig2.tif
A view of the inter­molecular *O*—*H*⋯Br/*O*—*H*⋯N_nitrile_ hydrogen bonding inter­actions (dashed lines) forming a helical one-dimensional chain, with displacement ellipsoids shown at the 50% probability level. See Table 1 for symmetry code (i). A thin solid line indicates an intra­molecular O—H⋯Br hydrogen bond, and a thick solid line indicates a π-stacking centroid-to-centroid inter­action.

CCDC reference: 1408281

Additional supporting information:  crystallographic information; 3D view; checkCIF report

## Figures and Tables

**Table 1:** Hydrogen-bond geometry (, )

*D*H*A*	*D*H	H*A*	*D* *A*	*D*H*A*
O1H1N1^i^	0.81(2)	2.04(2)	2.810(3)	159(2)
O1H1Br1*A*	0.81(2)	2.82(2)	3.262(5)	116(2)
O1H1Br1*A* ^i^	0.81(2)	2.62(2)	3.379(5)	156(2)

Symmetry code: (i) 
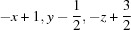
.

## supplementary crystallographic information

### S1. Structural commentary

The title compound, 3-bromo-2-hy­droxy­benzo­nitrile, may be prepared by the addition of a cyano group to *o*-bromo­phenol (Anwar & Hansen, 2008; Nakai *et al.* 2014). It has also recently been synthesized by the one-pot conversion of the salicylaldoxime, (*E*)-3-bromo-2-hy­droxy­benzaldehyde oxime, directly to 3-bromo-2-hy­droxy­benzo­nitrile (Whiting *et al.*, 2015). 3-Bromo-2-hy­droxy­benzo­nitrile is used as a synthetic reagent in the synthesis of 3,4-fused isoquinolin-1(2H)-one analogs (Li & Chua, 2011) and ampakine heterocycles which are a promising as a therapy for neurodegenerative diseases (Mulzer & Coates, 2011). The crystal structure of an isomer of the title compound which differs only in the position of the bromine substituent, 5-bromo-2-hy­droxy­benzo­nitrile, has previously been published (Oh & Tanski, 2012).

3-Bromo-2-hy­droxy­benzo­nitrile, (Fig. 1), crystallizes with a partial molecular packing disorder, where the bromine and nitrile substituents *ortho* to the phenol group are disordered with one another *via* a 180° rotation of the molecule about the carbon-oxygen bond of the phenol moiety. The disorder has been modeled as a disorder of only the bromine and nitrile substituents on a unique phenol ring, where the phenolic hydroxyl itself is not disordered, and the model has been refined with the help of similarity and advanced rigid bond restraints (Thorn *et al.*, 2012). Although the accuracy of the observed metrical parameters for the disordered groups is impacted by the refinement of the disorder, the bond lengths are nevertheless comparable to those found in related structures. The nitrile bond lengths C7—N1 and C7A—N1A of 1.161 (4) and 1.14 (2) Å, respectively, are similar to those seen in the related structures 5-bromo-2-hy­droxy­benzo­nitrile, with nitrile C≡N distance 1.142 (4) Å (Oh & Tanski, 2012), and the unbrominated analog, *o*-cyano­phenol, with C≡N distance 1.136 (2) Å (Beswick *et al.*, 1996). The aromatic bromine bond lengths C1—Br1A and C3—Br1 of 1.988 (5) Å and 1.907 (2) Å, respectively, are also similar to those seen in the related structure 5-bromo-2-hy­droxy­benzo­nitrile, with C—Br length 1.897 (3) Å (Oh & Tanski, 2012).

The molecules of the title compound pack together in the solid state with inter­molecular O—H···Br/O—H···N_nitrile_ hydrogen bonding (Fig. 2, Table 2). The hydrogen bonding is disordered with respect to the disordered bromine and nitrile substituents, not with respect to the phenol hydroxyl, which is found to have only one orientation. This hydrogen bonding forms a one-dimensional spiral chain extending parallel to the crystallographic *b*-axis, about the two-fold screw axis in P2_1_/c with direction [0,1,0] at 1/2, y, 1/4. Within the chains, the molecules also inter­act *via* an offset face-to-face π-stacking inter­action. This π-stacking is characterized by a centroid-to-centroid distance of 3.8488 (2) Å, a plane-to-centroid distance of 3.487 (1) Å, and a ring offset or ring-slippage distance of 1.630 (2) Å (Hunter & Saunders, 1990; Lueckheide *et al.*, 2013).

### S2. Synthesis and crystallization

3-Bromo-2-hy­droxy­benzo­nitrile (97%) was purchased from Aldrich Chemical Company, USA, and was recrystallized from acetone.

### S3. Refinement

The structure was refined against *F^2^* using all data with *SHELXL2014* (Sheldrick, 2015), employing established refinement strategies (Müller, 2009). All non-hydrogen atoms were refined anisotropically. Hydrogen atoms on carbon were included in calculated positions and refined using a riding model at C–H = 0.95 Å and *U*_iso_(H) = 1.2 × *U*_eq_(C) of the aryl C-atoms. Coordinates for the hydrogen atom on oxygen were taken from the difference Fourier synthesis and the hydrogen atom was subsequently refined semi-freely with the help of an O—H distance restraint (target value 0.84 (2) Å) while constraining its *U*_iso_ to 1.5 times the *U*_eq_ of the oxygen atom. The extinction parameter refined to zero and was removed from the refinement. The structure exhibits a partial molecular disorder. The disorder was successfully modeled and refined with the help of similarity restraints on 1,2- and 1,3-distances and displacement parameters as well as advanced rigid-bond restraints (Thorn *et al.*, 2012) for anisotropic displacement parameters, and inter­atomic distance restraints.

### Crystal data

**Table:**

C_7_H_4_BrNO	*F*(000) = 384
*M**_r_* = 198.02	*D*_x_ = 1.941 Mg m^−^^3^
Monoclinic, *P*2_1_/*c*	Mo *K*α radiation, λ = 0.71073 Å
*a* = 13.0171 (7) Å	Cell parameters from 6401 reflections
*b* = 3.8488 (2) Å	θ = 3.0–30.5°
*c* = 13.5989 (7) Å	µ = 5.98 mm^−^^1^
β = 96.062 (1)°	*T* = 125 K
*V* = 677.50 (6) Å^3^	Needle, colourless
*Z* = 4	0.22 × 0.10 × 0.04 mm

### Data collection

**Table:**

Bruker APEXII CCD diffractometer	1977 independent reflections
Radiation source: fine-focus sealed tube	1776 reflections with *I* > 2σ(*I*)
Graphite monochromator	*R*_int_ = 0.025
Detector resolution: 8.3333 pixels mm^-1^	θ_max_ = 30.0°, θ_min_ = 3.0°
φ and ω scans	*h* = −18→18
Absorption correction: multi-scan (*SADABS*; Bruker, 2013)	*k* = −5→5
*T*_min_ = 0.57, *T*_max_ = 0.80	*l* = −19→19
9903 measured reflections	

### Refinement

**Table:**

Refinement on *F*^2^	Primary atom site location: structure-invariant direct methods
Least-squares matrix: full	Secondary atom site location: difference Fourier map
*R*[*F*^2^ > 2σ(*F*^2^)] = 0.020	Hydrogen site location: mixed
*wR*(*F*^2^) = 0.049	H atoms treated by a mixture of independent and constrained refinement
*S* = 1.08	*w* = 1/[σ^2^(*F*_o_^2^) + (0.0241*P*)^2^ + 0.2328*P*] where *P* = (*F*_o_^2^ + 2*F*_c_^2^)/3
1977 reflections	(Δ/σ)_max_ = 0.002
110 parameters	Δρ_max_ = 0.37 e Å^−^^3^
102 restraints	Δρ_min_ = −0.45 e Å^−^^3^

### Special details

**Table:**

Geometry. All e.s.d.'s (except the e.s.d. in the dihedral angle between two l.s. planes) are estimated using the full covariance matrix. The cell e.s.d.'s are taken into account individually in the estimation of e.s.d.'s in distances, angles and torsion angles; correlations between e.s.d.'s in cell parameters are only used when they are defined by crystal symmetry. An approximate (isotropic) treatment of cell e.s.d.'s is used for estimating e.s.d.'s involving l.s. planes.
Refinement. Refinement of *F*^2^ against ALL reflections. The weighted *R*-factor *wR* and goodness of fit *S* are based on *F*^2^, conventional *R*-factors *R* are based on *F*, with *F* set to zero for negative *F*^2^. The threshold expression of *F*^2^ > σ(*F*^2^) is used only for calculating *R*-factors(gt) *etc*. and is not relevant to the choice of reflections for refinement. *R*-factors based on *F*^2^ are statistically about twice as large as those based on *F*, and *R*- factors based on ALL data will be even larger.

### Fractional atomic coordinates and isotropic or equivalent isotropic displacement parameters (Å^2^)

**Table:**

	*x*	*y*	*z*	*U*_iso_*/*U*_eq_	Occ. (<1)
O1	0.70927 (9)	0.4093 (4)	0.76425 (9)	0.0264 (3)	
H1	0.6483 (13)	0.420 (6)	0.7450 (18)	0.04*	
C1	0.66122 (12)	0.6091 (4)	0.92251 (12)	0.0195 (3)	
C7	0.5648 (2)	0.7338 (8)	0.8786 (2)	0.0225 (6)	0.9272 (13)
N1	0.4862 (2)	0.8421 (7)	0.84343 (18)	0.0281 (5)	0.9272 (13)
Br1A	0.5216 (4)	0.7707 (10)	0.8684 (3)	0.0332 (13)	0.0728 (13)
C2	0.73013 (11)	0.4575 (4)	0.86256 (11)	0.0185 (3)	
C3	0.82692 (11)	0.3564 (4)	0.90818 (11)	0.0186 (3)	
Br1	0.92397 (2)	0.15982 (5)	0.82834 (2)	0.02008 (6)	0.9272 (13)
C7A	0.8979 (14)	0.192 (6)	0.8577 (15)	0.02008 (6)	0.0728 (13)
N1A	0.9658 (13)	0.131 (5)	0.8138 (13)	0.02008 (6)	0.0728 (13)
C4	0.85334 (13)	0.3982 (4)	1.00854 (12)	0.0234 (3)	
H4	0.9194	0.3259	1.0376	0.028*	
C5	0.78350 (14)	0.5460 (5)	1.06728 (12)	0.0267 (3)	
H5	0.8017	0.5739	1.1363	0.032*	
C6	0.68739 (13)	0.6519 (4)	1.02446 (12)	0.0237 (3)	
H6	0.6394	0.7532	1.064	0.028*	

### Atomic displacement parameters (Å^2^)

**Table:**

	*U*^11^	*U*^22^	*U*^33^	*U*^12^	*U*^13^	*U*^23^
O1	0.0175 (5)	0.0414 (7)	0.0194 (5)	0.0021 (5)	−0.0026 (4)	−0.0046 (5)
C1	0.0174 (7)	0.0191 (7)	0.0216 (7)	−0.0012 (5)	0.0008 (6)	0.0021 (6)
C7	0.0186 (12)	0.0255 (11)	0.0237 (10)	0.0015 (11)	0.0044 (11)	−0.0002 (7)
N1	0.0217 (12)	0.0369 (13)	0.0253 (11)	0.0038 (9)	0.0012 (8)	0.0005 (9)
Br1A	0.029 (3)	0.031 (2)	0.040 (2)	0.001 (2)	0.003 (2)	−0.0004 (15)
C2	0.0173 (7)	0.0186 (7)	0.0190 (7)	−0.0024 (6)	−0.0007 (5)	0.0016 (6)
C3	0.0161 (6)	0.0173 (7)	0.0224 (7)	−0.0009 (6)	0.0012 (5)	0.0015 (6)
Br1	0.01502 (9)	0.02106 (9)	0.02437 (10)	0.00176 (7)	0.00305 (6)	−0.00176 (7)
C7A	0.01502 (9)	0.02106 (9)	0.02437 (10)	0.00176 (7)	0.00305 (6)	−0.00176 (7)
N1A	0.01502 (9)	0.02106 (9)	0.02437 (10)	0.00176 (7)	0.00305 (6)	−0.00176 (7)
C4	0.0198 (7)	0.0252 (8)	0.0241 (8)	−0.0006 (6)	−0.0037 (6)	0.0047 (6)
C5	0.0290 (8)	0.0325 (9)	0.0176 (7)	−0.0004 (7)	−0.0023 (6)	0.0027 (7)
C6	0.0236 (8)	0.0265 (8)	0.0215 (7)	−0.0004 (6)	0.0043 (6)	0.0003 (7)

### Geometric parameters (Å, º)

**Table:**

O1—C2	1.3487 (19)	C3—C4	1.381 (2)
O1—H1	0.810 (16)	C3—Br1	1.9071 (16)
C1—C2	1.401 (2)	C7A—N1A	1.143 (16)
C1—C6	1.402 (2)	C4—C5	1.393 (2)
C1—C7	1.416 (3)	C4—H4	0.95
C1—Br1A	1.988 (5)	C5—C6	1.384 (2)
C7—N1	1.161 (4)	C5—H5	0.95
C2—C3	1.400 (2)	C6—H6	0.95
C3—C7A	1.363 (14)		
			
C2—O1—H1	113.7 (18)	C4—C3—Br1	119.86 (12)
C2—C1—C6	121.38 (14)	C2—C3—Br1	118.52 (11)
C2—C1—C7	119.29 (17)	N1A—C7A—C3	164 (3)
C6—C1—C7	119.28 (17)	C3—C4—C5	120.30 (15)
C2—C1—Br1A	122.09 (17)	C3—C4—H4	119.9
C6—C1—Br1A	116.52 (16)	C5—C4—H4	119.9
N1—C7—C1	178.7 (4)	C6—C5—C4	119.63 (15)
O1—C2—C3	118.60 (14)	C6—C5—H5	120.2
O1—C2—C1	124.04 (14)	C4—C5—H5	120.2
C3—C2—C1	117.35 (14)	C5—C6—C1	119.71 (16)
C7A—C3—C4	116.1 (9)	C5—C6—H6	120.1
C7A—C3—C2	122.2 (9)	C1—C6—H6	120.1
C4—C3—C2	121.62 (15)		
			
C6—C1—C2—O1	179.94 (15)	C1—C2—C3—Br1	−178.46 (11)
C7—C1—C2—O1	−2.6 (3)	C4—C3—C7A—N1A	−88 (8)
Br1A—C1—C2—O1	0.4 (3)	C2—C3—C7A—N1A	96 (8)
C6—C1—C2—C3	−1.2 (2)	C7A—C3—C4—C5	−176.5 (12)
C7—C1—C2—C3	176.33 (18)	C2—C3—C4—C5	−0.4 (2)
Br1A—C1—C2—C3	179.33 (18)	Br1—C3—C4—C5	179.11 (13)
O1—C2—C3—C7A	−4.1 (13)	C3—C4—C5—C6	−0.2 (3)
C1—C2—C3—C7A	176.9 (13)	C4—C5—C6—C1	0.1 (3)
O1—C2—C3—C4	−179.97 (15)	C2—C1—C6—C5	0.6 (2)
C1—C2—C3—C4	1.1 (2)	C7—C1—C6—C5	−176.89 (19)
O1—C2—C3—Br1	0.5 (2)	Br1A—C1—C6—C5	−179.86 (18)

### Hydrogen-bond geometry (Å, º)

**Table:**

*D*—H···*A*	*D*—H	H···*A*	*D*···*A*	*D*—H···*A*
O1—H1···N1^i^	0.81 (2)	2.04 (2)	2.810 (3)	159 (2)
O1—H1···Br1*A*	0.81 (2)	2.82 (2)	3.262 (5)	116 (2)
O1—H1···Br1*A*^i^	0.81 (2)	2.62 (2)	3.379 (5)	156 (2)

Symmetry code: (i) −*x*+1, *y*−1/2, −*z*+3/2.
