# Supplementary figures and images for: Crystal structure of 3-bromo-2-hy­droxy­benzo­nitrile
Source: Acta Crystallogr E Crystallogr Commun. 2015 Jun 27;71(Pt 7):o523–4. doi: 10.1107/S2056989015011974 (PMC4518954; doi:10.1107/S2056989015011974)

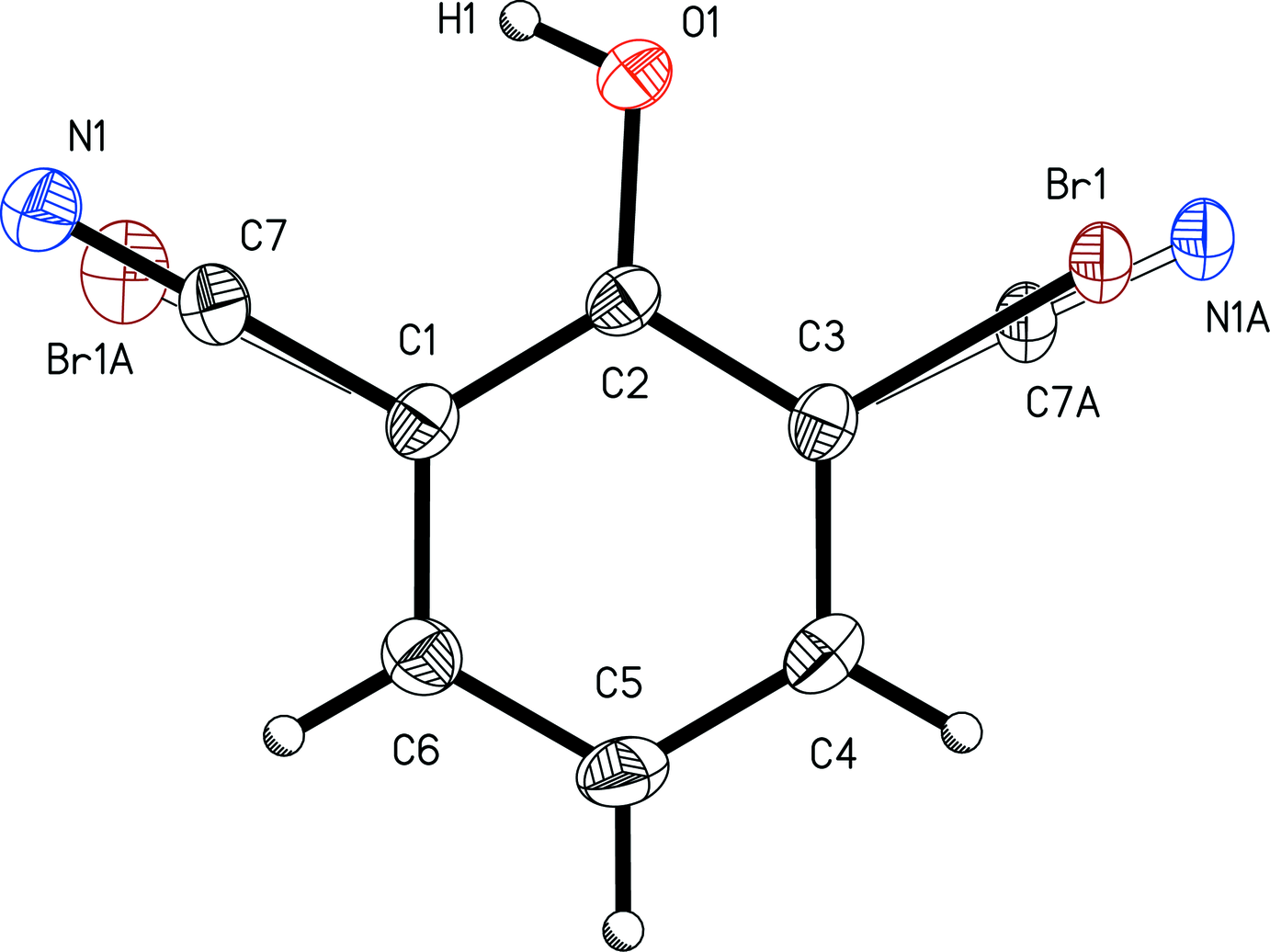

Supplement: Supplementary file 4 [file e-71-0o523-fig1.tif]

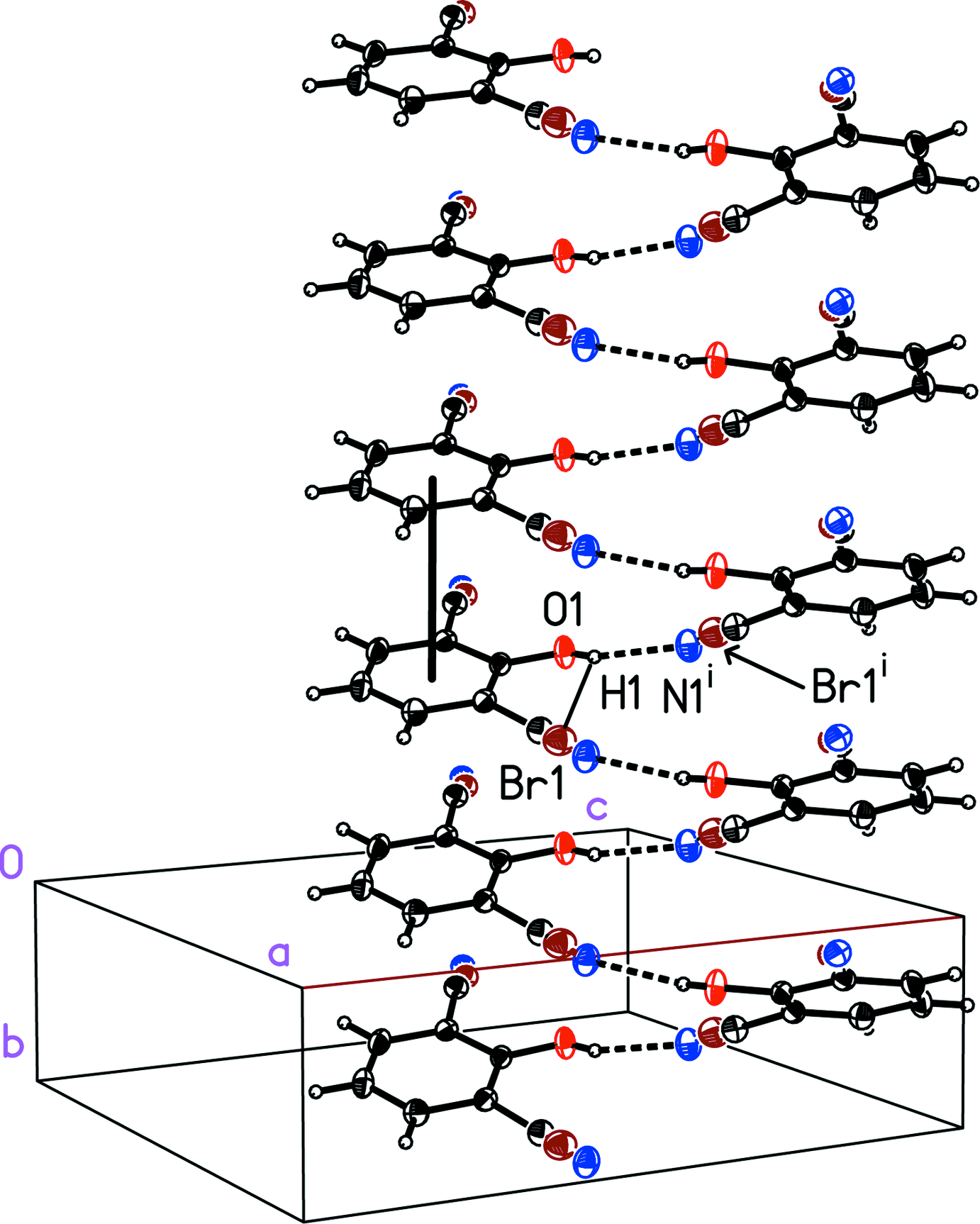

Supplement: Supplementary file 5 [file e-71-0o523-fig2.tif]
